# Dietary allyl-isothiocyanate affects male triglyceride levels in *Drosophila melanogaster* without detectable changes in microbiota composition

**DOI:** 10.3389/fmicb.2026.1817451

**Published:** 2026-05-13

**Authors:** Sonja Dähn, Sabine Hurka, Daniel Kreft, Juan Guzman, Anja Poehlein, Andreas Vilcinskas, Dorothee Tegtmeier, Anika E. Wagner

**Affiliations:** 1 Institute of Nutritional Science, Justus Liebig University Giessen, Giessen, Germany; 2 BMFTR Junior Research Group in Bioeconomy (BioKreativ) “SymBioÖkonomie”, Fraunhofer Institute for Molecular Biology and Applied Ecology (IME), Giessen, Germany; 3 Branch for Bioresources, Fraunhofer Institute for Molecular Biology and Applied Ecology (IME), Giessen, Germany; 4 Institute of Microbiology and Genetics, University of Göttingen, Göttingen, Germany; 5 Institute of Insect Biotechnology, Justus Liebig University Giessen, Giessen, Germany; 6 Center for Sustainable Food Systems, Justus Liebig University Giessen, Giessen, Germany

**Keywords:** allyl-isothiocyanate (AITC), *Drosophila melanogaster*, gut microbiota, lipid metabolism, sex-specific metabolic effects

## Abstract

Allyl-isothiocyanate (AITC), a bioactive compound derived from glucosinolates in cruciferous vegetables, is known for its antimicrobial, anti-inflammatory, and metabolic effects in mammals. However, its *in vivo* interaction with the gut microbiota and consequent physiological outcomes remain poorly understood. Here, we investigated the impact of dietary AITC on host metabolism and microbiota composition in *Drosophila melanogaster*, a well-established model for nutritional and metabolic research. Male and female flies were fed a standard diet supplemented with 0.25 mM AITC, with or without antibiotic treatment, for 10 and 30 days. Physiological parameters (body weight, glucose, triglycerides, and survival) were measured, and microbial community profiles were analyzed via 16S rRNA gene amplicon sequencing. AITC supplementation did not significantly affect body weight, glucose content, or survival, regardless of sex or antibiotic co-treatment. However, after 30 days, a significant reduction in triglyceride levels was observed in male flies exposed to AITC (*p* < 0.05), suggesting a sex-specific metabolic response that persisted under antibiotic treatment. Analysis of the microbiota revealed that the dominant bacterial classes were *Alphaproteobacteria*, *Bacilli*, and *Gammaproteobacteria*, together comprising approximately 77.7% of all detected amplicon sequence variants. AITC did not alter microbial alpha or beta diversity, whereas age and sex significantly influenced community composition. Notably, alpha diversity decreased in older flies. These findings indicate that AITC at the tested concentration does not alter the microbiota in *D. melanogaster* but may induce sex-dependent effects on lipid metabolism. The absence of clear effects on the microbiota suggests that the observed physiological actions *in vivo* are not associated with detectable changes in microbiota composition. urn:lsid:zoobank.org:act:5B39F0AA-270D-4AA8-B9A3-C36A3A265910. Drosophila melanogaster, Meigen 1830.

## 1 Introduction

The human digestive tract is home to an enormous number of microorganisms, including bacteria, archaea, and fungi. This collective is known as the gut microbiota (Rinninella et al., 2019; Siddiqui and Cresci, 2021) which plays a central role in human health, regulating many aspects of human physiology, immunity and behavior (Cryan and Dinan, 2012; Belkaid and Hand, 2014; Honda and Littman, 2016; Fung et al., 2017). Alterations in the composition of the gut microbiota have been associated with different diseases, such as type 2 diabetes, although causal relationships remain unclear (Larsen et al., 2010; Walsh et al., 2014).

Allyl-isothiocyanate (AITC), a bioactive plant compound responsible for the pungent taste of, for example, mustard, radish or wasabi, is generated from its precursor sinigrin by enzymatic degradation catalyzed by myrosinase (Kim et al., 2015). It has been reported to exhibit several health-promoting effects in humans, including antioxidant and anti-inflammatory effects, as well as antimicrobial and anticancer properties (Lin et al., 2000; Romeo et al., 2018; Tran et al., 2021; Tarar et al., 2022). In addition, AITC has been associated with antidiabetic properties (Ahn et al., 2014; Lo et al., 2018; Sahin et al., 2019; Okulicz et al., 2021). In *in vitro* approaches AITC inhibited the growth of different bacteria, including *Campylobacter jejuni*, *Escherichia coli*, *Listeria monocytogenes* and *Clostridium perfringens* (Lin et al., 2000; Dufour et al., 2012; Borges et al., 2015; Li et al., 2023). Despite these findings, information on the impact of AITC on gut microbiota is scarce. In 2025, an *in vitro* colonic fermentation in the presence of AITC was performed, resulting in an increased alpha diversity (Cámara-Martos et al., 2025). As the gut microbiota has a significant impact on human health, AITC might be able to mediate health-promoting properties through changing the composition of the intestinal microbiota. In addition, these changes may also alter AITC metabolism since gut microorganisms might play a key role in AITC metabolism.

The fruit fly *Drosophila melanogaster* is an important model organism and especially known for its use in genetics. Since important signaling pathways orchestrating for example energy metabolism (insulin signaling) or immunity (toll signaling) are similar between humans and *D. melanogaster*, the fruit fly is increasingly used as a model organism in medical and nutritional research (Droujinine and Perrimon, 2016; Staats et al., 2018a; Baenas and Wagner, 2019; Dähn and Wagner, 2025). As axenic fruit flies can be generated relatively easily, it has been employed to elucidate intestinal host-bacteria interactions (Heys et al., 2018; Capo et al., 2019; Ludington and Ja, 2020; Chiang et al., 2022). Although our group demonstrated that AITC can modulate immune responses in *D. melanogaster*, particularly in the context of infection (Zimmermann et al., 2024), its effects under chronic dietary exposure, as well as potential interactions with the gut microbiota, remain poorly understood. While data of the impact of AITC on colonic fermentation are available (Cámara-Martos et al., 2025), *in vivo* studies investigating a potential impact of AITC on the gut microbiota are currently lacking. Given that AITC may exert antimicrobial effects and affect host metabolism, a better understanding of mechanisms underlying AITC’s interaction with the gut microbiota *in vivo* could provide valuable insights. We therefore hypothesized that a dietary AITC supplementation affects the fly’s gut microbiota and in consequence the host’s physiology. Hence, we fed *D. melanogaster* AITC-containing feed with and without antibiotic treatment for 10 and 30 days, respectively, determined the body composition reflected in glucose and triglyceride levels and analyzed the fly’s intestinal microbiota.

## 2 Materials and methods

### 2.1 Fly husbandry

For all experiments the *D. melanogaster* strain w^1118^ (Bloomington Drosophila Stock Center #5905, Indiana, USA) was used. Flies were maintained at 25 °C and 60% humidity under a 12/12 h light/dark cycle in an incubator (HPP 1018, Memmert, Schwalbach, Germany) on standard Caltech (CT) medium. For experiments, 3-day-old (to ensure, all flies were mated) age-matched flies from synchronized eggs were immobilized on ice and separated according to their sex. Female and male flies were transferred to separate vials containing a diet with 10% sugar (SY10 medium) under four treatment conditions: control, AITC only, antibiotics only and AITC combined with antibiotics. Each treatment group consisted of three vials containing 25 flies. CT medium consists of 5.5% dextrose, 3.0% sucrose (Carl Roth, Karlsruhe, Germany), 6.0% corn meal, 2.5% inactive dry yeast, 1.0% agar, 0.3% Tegosept (Kisker, Steinfurt, Germany), and 0.3% propionic acid (Carl Roth, Karlsruhe, Germany). SY10 medium comprises 10% sucrose, 10% inactive dry yeast, 2.0% agar, 0.3% Tegosept (Kisker, Steinfurt, Germany), and 0.3% propionic acid (Carl Roth, Karlsruhe, Germany).

### 2.2 Allyl-isothiocyanate and antibiotic treatment

For AITC treatment, 1 M stock solution of AITC (Sigma-Aldrich, Taufkirchen, Germany) was prepared in ethanol (abs.) (Merck, Darmstadt, Germany) and added to the SY10 medium to reach a final concentration of 0.25 mM. The control medium contained the same amount of ethanol (abs.) as the solvent control. Antibiotic treatment was performed as described in Staats et al. (2018b). To eliminate the gut microbiota, 500 μg/mL ampicillin sodium salt (Carl Roth, Karlsruhe, Germany), 50 μg/mL tetracycline hydrochloride (Merck, Darmstadt, Germany), and 200 μg/mL rifamycin sodium salt (Merck, Darmstadt, Germany) were added to the experimental feed.

### 2.3 Survival rate and body composition

AITC and antibiotic treatment may have an impact on the flies’ survival rates. To assess this, the lifespan was recorded over 30 days. The flies were sorted and housed as described above. Every second-third day, the flies were transferred to a new vial with fresh feed containing AITC, antibiotics, a combination of antibiotics and AITC or control feed and the number of dead flies was recorded.

To obtain an overview of the influence of AITC on energy metabolism, we also measured the flies’ body weight, triglyceride and glucose content. Therefore, 25 age-matched flies were maintained as described above for 10 or 30 days with or without AITC/antibiotics. Then, flies were weighed and frozen at −80 °C until further use. For measurements, 5 flies from each vial were homogenized in 250 μL PBS with 1% Triton X in a Tissue Lyzer II (Qiagen, Hilden, Germany) and centrifuged at 5,000 *g* for 10 min. The supernatant was transferred into a fresh vial and stored at −20 °C until further use. To determine the glucose and triglyceride content a Glucose-Kit (Dialab, Neudorf, Austria) and a Triglyceride-Kit (Dialab, Neudorf, Austria) was used, following the manufacturer’s instructions. The glucose and triglyceride contents were normalized to the mean contents of the respective control group for the calculation.

### 2.4 Sample preparation for microbial community analysis

To investigate the impact of AITC on microbial communities in *D. melanogaster,* we used 16S rRNA gene amplicon sequencing. Treatments with SY10 control and AITC were used for this approach. After treatment for 10 or 30 days, 5 flies per vial were pooled and frozen at −80 °C for further analysis. Flies were not surface sterilized prior to DNA extraction, as the aim was to characterize the total microbial community associated with the organism, including both internal and external (cuticular) bacteria. The gut was dissected from 5 additional flies per vial under a stereomicroscope with sterile forceps, pooled and stored in sterile PBS at −80 °C until further use. Six replicate vials for each experimental group were used (96 samples in total). DNA from whole *D. melanogaster* individuals and dissected guts was extracted using the NucleoSpin Soil Kit (Macherey-Nagel, Düren, Germany) according to the manufacturer’s instructions with an additional bead beating step for two times at 5 m s^−1^ for 30 s (FastPrep-24, MP Biomedicals, Solon, OH, United States). DNA was eluted in a final volume of 30 μL. The V3–V4 region of the 16S rRNA gene was amplified using primers 5′-CCTACGGGNGGCWGCAG and 3′-GACTACHVGGGTATCTAATCC (Klindworth et al., 2013), along with Illumina adapter for library preparation. Each DNA sample was amplified in triplicate (Guzman et al., 2022) using 50 μL reactions containing 25 μL Phusion Flash High-Fidelity PCR Master Mix (New England Biolabs, Ipswich, MA, United States), 20 μL nuclease-free water, 2 μL of each primer, and 1 μL of undiluted DNA template. The thermal cycling program was as follows: initial denaturation at 98 °C for 1 min, followed by 30 cycles of 98 °C for 45 s, 60 °C for 45 s, and 72 °C for 30 s and a final extension at 72 °C for 5 min. Library preparation was verified by agarose gel electrophoresis. The triplicates were pooled evenly to a total of 120 μL and size-selected using the GeneRead Size Selection Kit (Qiagen, Hilden, Germany) following the manufacturer’s quick-start protocol (50 μL per sample) and quantified spectrophotometrically on a Take 3 plate reader (BioTek Instruments, Winooski, VT, United States). Sequencing was performed on an Illumina MiSeq platform at the Genomics Laboratory of Georg-August University Göttingen (G2L, Göttingen, Germany) generating paired-end reads with a read length of 300 base pairs.

### 2.5 Bioinformatic processing and microbiota profiling

Demultiplexing the sequencing reads was conducted at G2L prior to analysis. All downstream processing was performed using the QIIME2 version 2025.7 amplicon distribution (Bolyen et al., 2019). Primers were clipped using the cutadapt plugin (Martin, 2011), followed by denoising and merging reads, filtering for chimeric sequences and generating amplicon sequence variants (ASV) with the DADA2 plugin (Callahan et al., 2016). The RESCRIPt plugin (Robeson et al., 2021) was used to construct a self-trained naïve Bayes classifier based on SILVA 138.2 with a sequence identity threshold of 99% (Quast et al., 2013) with reference sequences trimmed to the target region before (Werner et al., 2012). Taxonomic classification was performed with a minimum confidence threshold of 0.7 (Bokulich et al., 2018). Non-target ASVs and all sequences classified as genus *Wolbachia* as a common endosymbiont in *D. melanogaster* were removed to focus on the remaining microbial community composition. All ASVs were classified to the lowest feasible taxonomic rank, but results are reported at the class and family level because of the limitations of amplicon sequencing. Alpha diversity metrics [Pielou’s evenness, Shannon index, species richness, and Faith’s Phylogenetic Diversity (PD)] and beta diversity metrics (weighted UniFrac distance) were computed with the core-metrics-phylogenetic plugin using a rarefied sampling depth of 6,802 reads.

### 2.6 Statistical analysis

To compare the results of the survival rate, the body weight and the triglyceride and glucose contents of the flies a one-way ANOVA followed by Dunnett’s multiple comparison *post hoc* test was performed using GraphPad Prism 10 (GraphPad Software, Boston, MA, United States) with significance accepted at *p* < 0.05. Due to well-established baseline differences between sexes and age groups, statistical analyses were primarily performed within each group to assess treatment-specific effects. Direct comparisons between sexes or age groups were not the focus of this study, instead the study aimed to determine whether AITC induces changes under each condition and whether such effects depend on age or sex.

All statistical analyses regarding the microbial analysis were performed in R 4.5 (R Core Team, 2025) using the packages qiime2R (Bisanz, 2018), tidyverse (Wickham et al., 2019), ggh4x (van den Brand, 2025), vegan (Oksanen et al., 2025), and plyr (Wickham, 2011). For alpha diversity, data distributions were tested for normality using the Shapiro–Wilk test. Since all metrics were non-normally distributed, Kruskal–Wallis tests followed by Dunn’s post hoc tests with Benjamini–Hochberg (BH) adjustment (adj.) were applied. When analyzing multiple metrics, we first collected all Dunn results without adjusting the *p*-values and then applied the BH adjustment to all collected values in a single step. Beta diversity was assessed using weighted UniFrac distance metric. Group dispersion homogeneity was evaluated using the betadisper function in vegan, followed by PERMANOVA (Permutational Multivariate Analysis of Variance) using the adonis2 function in the same package (9,999 permutations; p-value adjustment with BH).

## 3 Results

### 3.1 Survival and body composition

**Figure 1:**
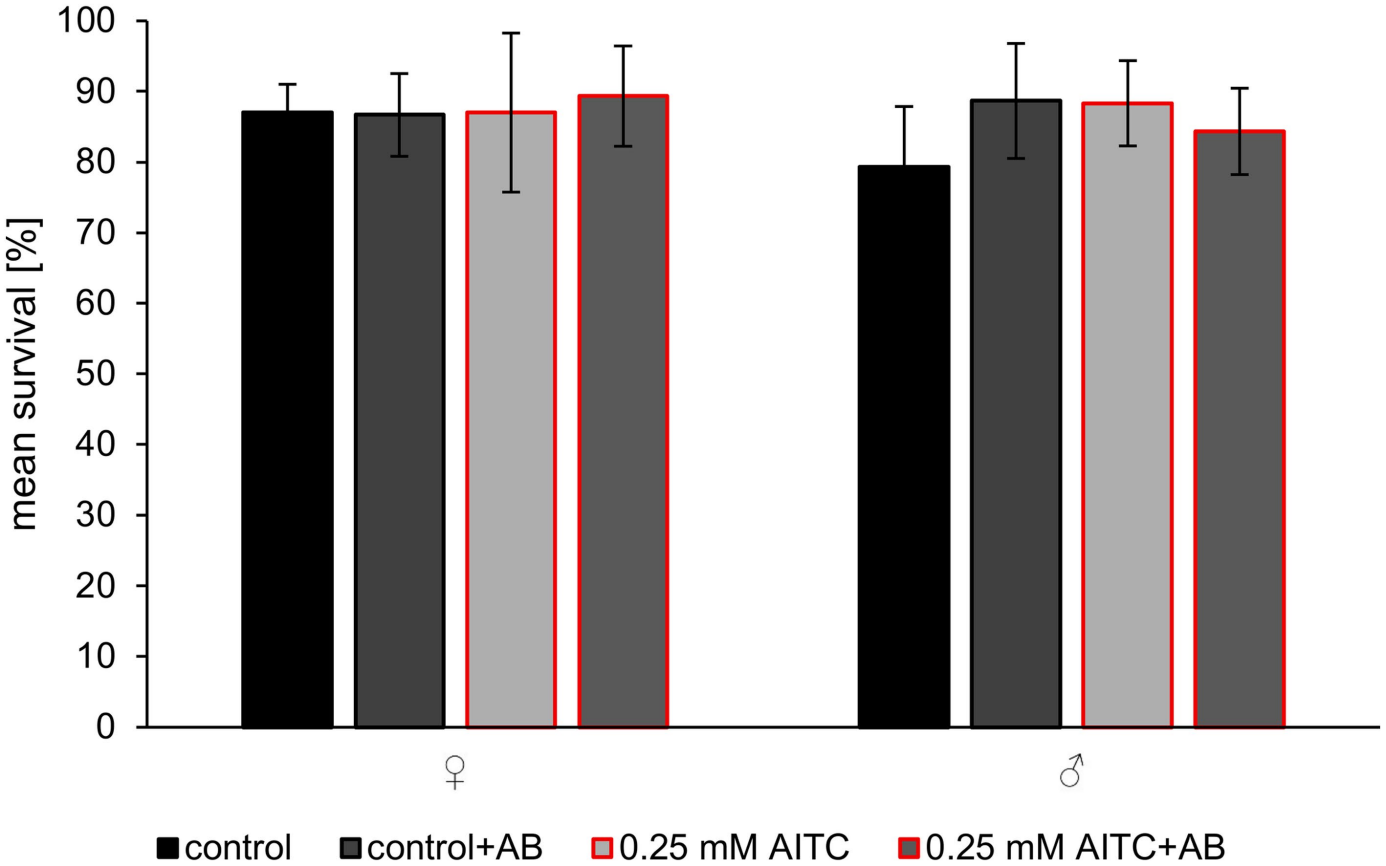
Mean survival of female (♀) and male (♂) *Drosophila melanogaster*, after 30 days of treatment with or without 0.25 mM AITC and antibiotics (AB, bar outlined in red). Flies were maintained under standard conditions and subjected to either control diet, 0.25 mM AITC, antibiotic treatment (AB: 500 μg/ml ampicillin, 50 μg/mL tetracycline, and 200 μg/mL rifamycin), or a combination of both (0.25 mM AITC + AB). Data are presented as mean ± SD of three independent experiments (*n* = 3) at different timepoints, each consisting of 75 flies. Statistical analysis was performed using a one-way ANOVA followed by Dunnett’s multiple comparison *post hoc* test.

We first investigated the impact of AITC and antibiotic treatment on survival rate. Survival assays revealed that neither AITC nor antibiotic treatment affected survival after 30 days of treatment (Figure 1). Both female and male flies exhibited comparable mean survival rates across all treatment groups, including the exposure to a combination of AITC and antibiotics (control vs. 0.25 mM AITC: *p* = 0.99; control vs. control + AB: *p* = 0.99 and control vs. 0.25 mM AITC + AB: *p* = 0.96). Kaplan–Meier survival analysis also revealed no significant differences between groups (Supplementary Figure S1).

**Figure 2:**
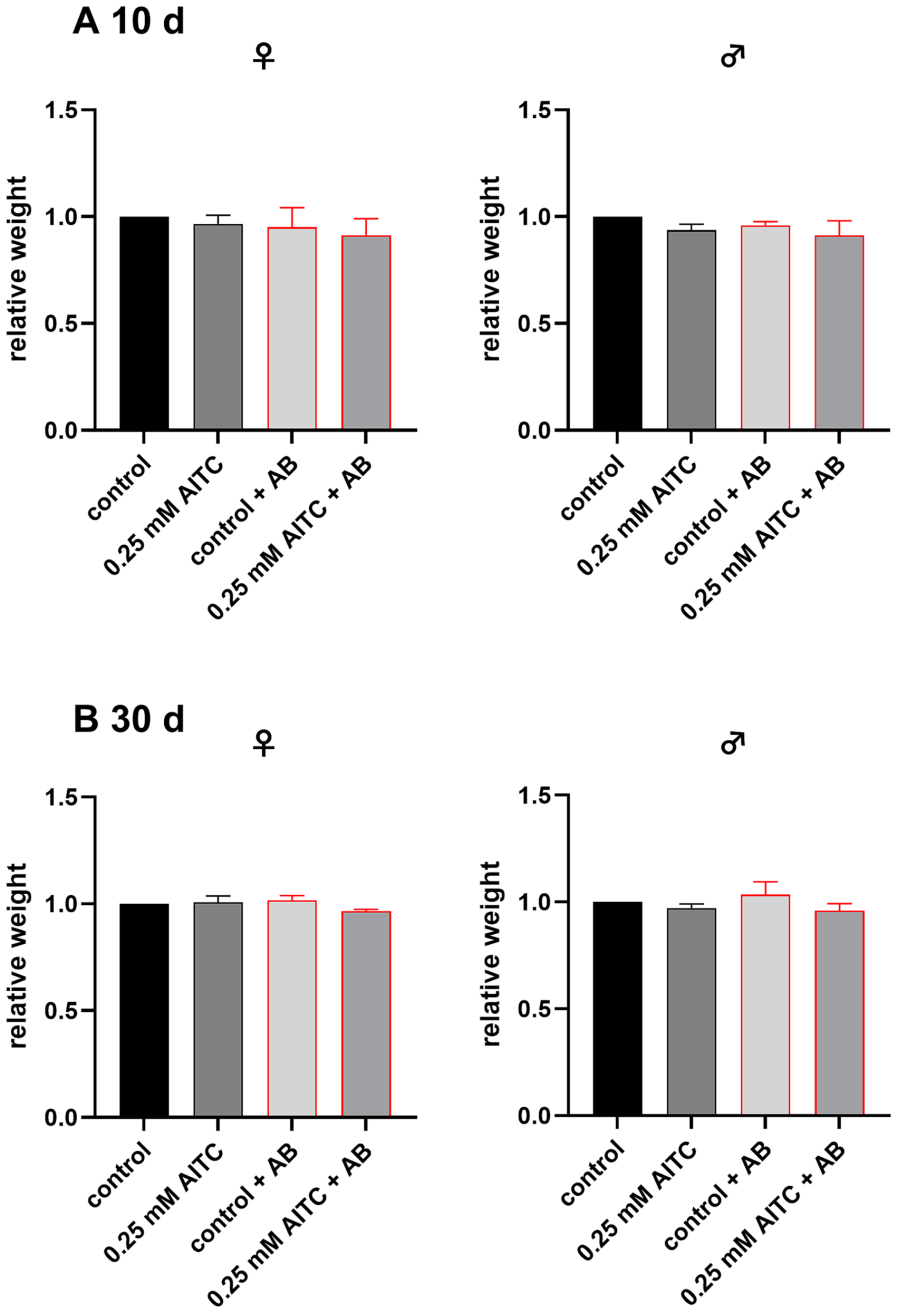
Relative weight of female (♀) and male (♂) *Drosophila melanogaster*, after 10 **(A)** and 30 **(B)** days of treatment with or without 0.25 mM AITC and antibiotics (AB, bar outlined in red). Flies were maintained under standard conditions and subjected to either control diet, 0.25 mM AITC, antibiotic treatment (AB: 500 µg/mL ampicillin, 50 μg/mL tetracycline, and 200 μg/mL rifamycin), or a combination of both (0.25 mM AITC + AB). Body weight was normalized to the respective control group. Data are presented as mean + SD of three independent experiments (*n* = 3) at different timepoints, each consisting of 5 flies. Statistical analysis was performed using a one-way ANOVA followed by Dunnett’s multiple comparison *post hoc* test.

As AITC is known to affect energy metabolism (Ahn et al., 2014; Lo et al., 2018; Sahin et al., 2019; Okulicz et al., 2021), we investigated the effect of AITC and antibiotic treatment on the flies’ weight. As shown in Figure 2, the body weight was statistically consistent irrespective of sex, age (10-day female flies: control vs. 0.25 mM AITC: *p* = 0.84; control vs. control + AB: *p* = 0.67; control vs. 0.250 mM AITC + AB: *p* = 0.29; 10-day male flies: control vs. 0.25 mM AITC: *p* = 0.17; control vs. control + AB: *p* = 0.46; control vs. 0.250 mM AITC + AB: *p* = 0.052 and 30-day old female flies control vs. 0.25 mM AITC: *p* = 0.94; control vs. control + AB: *p* = 0.59; control vs. 0.250 mM AITC + AB: *p* = 0.13; 30-day male flies: control vs. 0.25 mM AITC: *p* = 0.67; control vs. control + AB: *p* = 0.52; control vs. 0.250 mM AITC + AB: *p* = 0.4) and across the different treatment groups.

**Figure 3:**
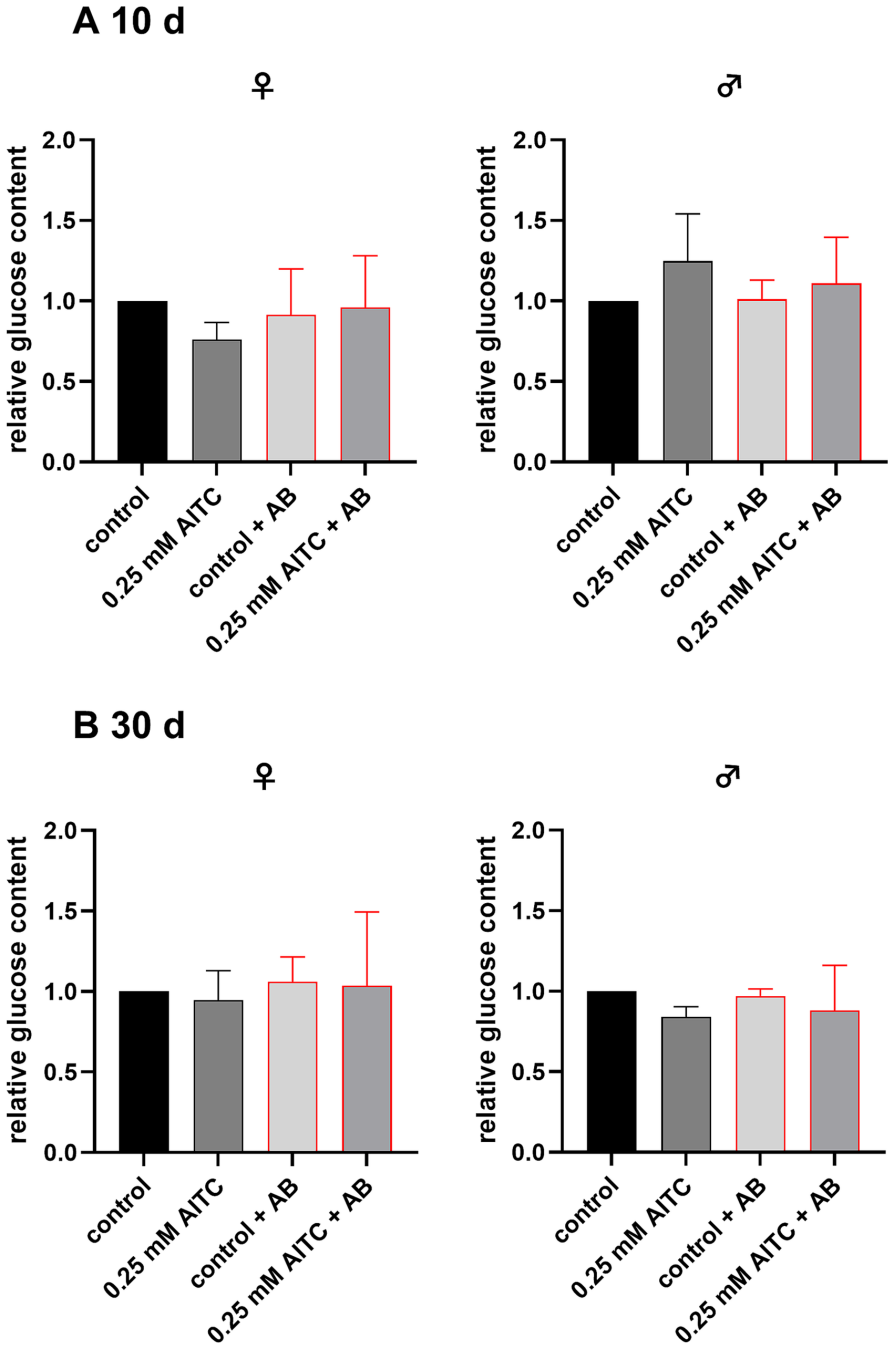
Relative glucose content of female (♀) and male (♂) *Drosophila melanogaster*, after 10 **(A)** and 30 **(B)** days of treatment with or without 0.25 mM AITC and antibiotics (AB, bar outlined in red). Flies were maintained under standard conditions and subjected to either control diet, 0.25 mM AITC, antibiotic treatment (AB: 500 μg/ml ampicillin, 50 μg/mL tetracycline, and 200 μg/mL rifamycin), or a combination of both (0.25 mM AITC + AB). Glucose content was normalized to the respective control group. Data are presented as mean + SD of three independent experiments (*n* = 3) at different timepoints, each consisting of 5 flies. Statistical analysis was performed using a one-way ANOVA followed by Dunnett’s multiple comparison *post hoc* test.

To obtain deeper insights into energy metabolism, we measured the specific glucose and triglyceride levels in our treated *D. melanogaster*. As presented in Figure 3 the relative glucose content of *D. melanogaster* did not differ significantly between treatment groups. Neither AITC nor antibiotic treatment, alone or in combination, affected glucose content in females and males after 10 days or 30 days (10-day female flies: control vs. 0.25 mM AITC: *p* = 0.45; control vs. control + AB: *p* = 0.93; control vs. 0.250 mM AITC + AB: *p* = 0.99; 10-day male flies: control vs. 0.25 mM AITC: *p* = 0.4; control vs. control + AB: *p* = 0.99; control vs. 0.250 mM AITC + AB: *p* = 0.86 and 30-day old female flies control vs. 0.25 mM AITC: p = 0.99; control vs. control + AB: *p* = 0.98; control vs. 0.250 mM AITC + AB: p = 0.99; 30-day male flies: control vs. 0.25 mM AITC: *p* = 0.44; control vs. control + AB: p = 0.99; control vs. 0.250 mM AITC + AB: *p* = 0.64).

**Figure 4:**
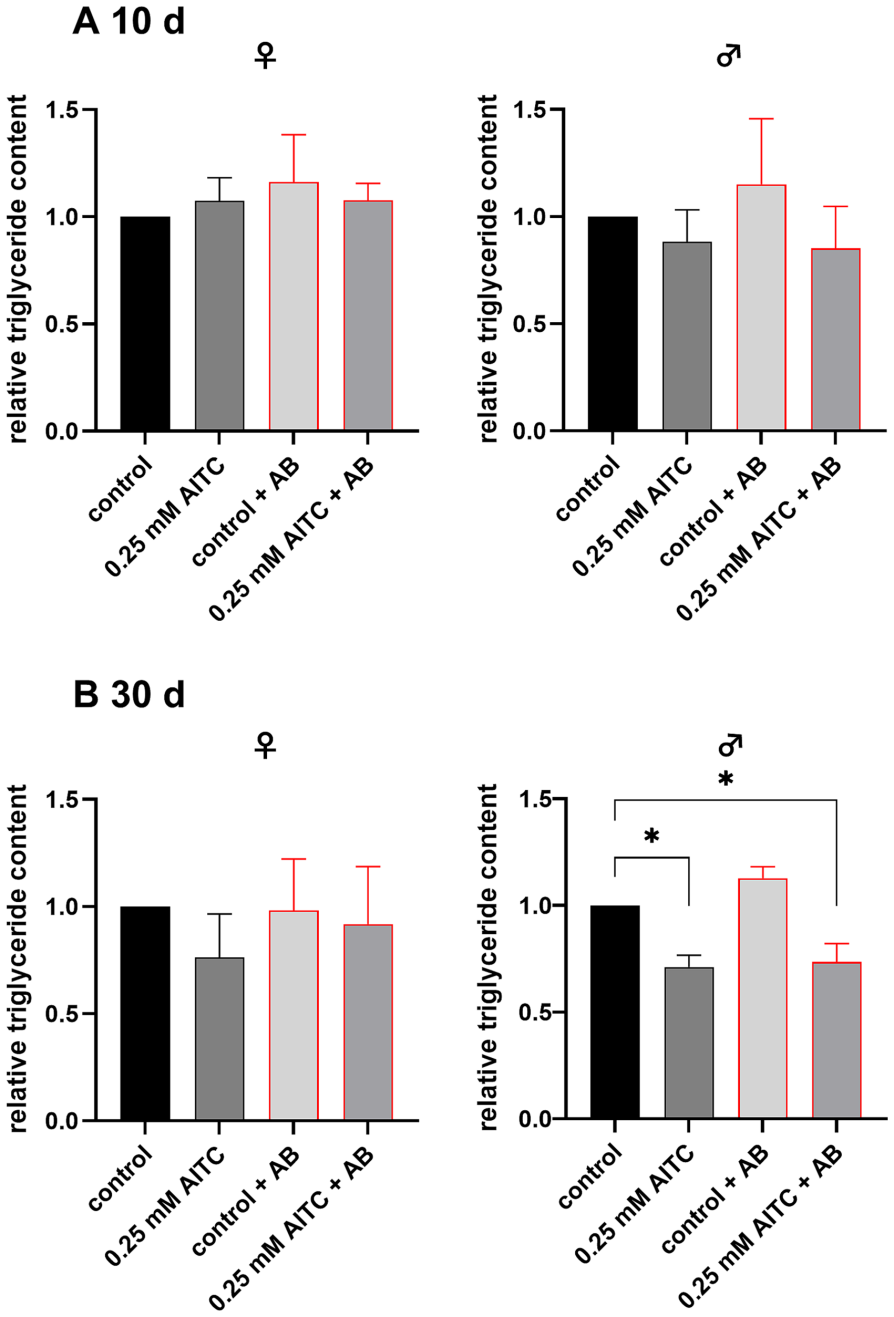
Relative triglyceride content of female (♀) and male (♂) *Drosophila melanogaster*, after 10 **(A)** and 30 **(B)** days of treatment with or without 0.25 mM AITC and antibiotics (AB, bar outlined in red). Flies were maintained under standard conditions and subjected to either control diet, 0.25 mM AITC, antibiotic treatment (AB: 500 μg/mL ampicillin, 50 μg/mL tetracycline, and 200 μg/mL rifamycin), or a combination of both (0.25 mM AITC + AB). Triglyceride content was normalized to the respective control group. Data are presented as mean + SD of three independent experiments (*n* = 3) at different timepoints, each consisting of 5 flies. Statistical analysis was performed using a one-way ANOVA followed by Dunnett’s multiple comparison post hoc test. ^*^p < 0.05.

As shown in Figure 4, the relative triglyceride content differed depending on sex and treatment duration. After 10 days, no significant differences in triglyceride levels were detected between treatment groups, neither in female nor in male flies (Figure 4A; female flies: control vs. 0.25 mM AITC: *p* = 0.82; control vs. control + AB: *p* = 0.35; control vs. 0.250 mM AITC + AB: *p* = 0.81; male flies: control vs. 0.25 mM AITC: *p* = 0.81; control vs. control + AB: *p* = 0.68; control vs. 0.250 mM AITC + AB: *p* = 0.69). However, after 30 days, a significant reduction in triglyceride content was observed in males treated with 0.25 mM AITC compared to the control group (Figure 4B, *p* = 0.028). Whereas antibiotic treatment alone had no effect on triglyceride content (*p* = 0.0645), the combination of AITC and antibiotic treatment also significantly lowered the triglyceride levels in males (*p* = 0.045), while no significant changes were detected in females (control vs. 0.25 mM AITC: *p* = 0.41; control vs. control + AB: *p* = 0.99; control vs. 0.250 mM AITC + AB: *p* = 0.93). For absolute values see Supplementary Figure S2.

### 3.2 Microbial community analysis

**Figure 5:**
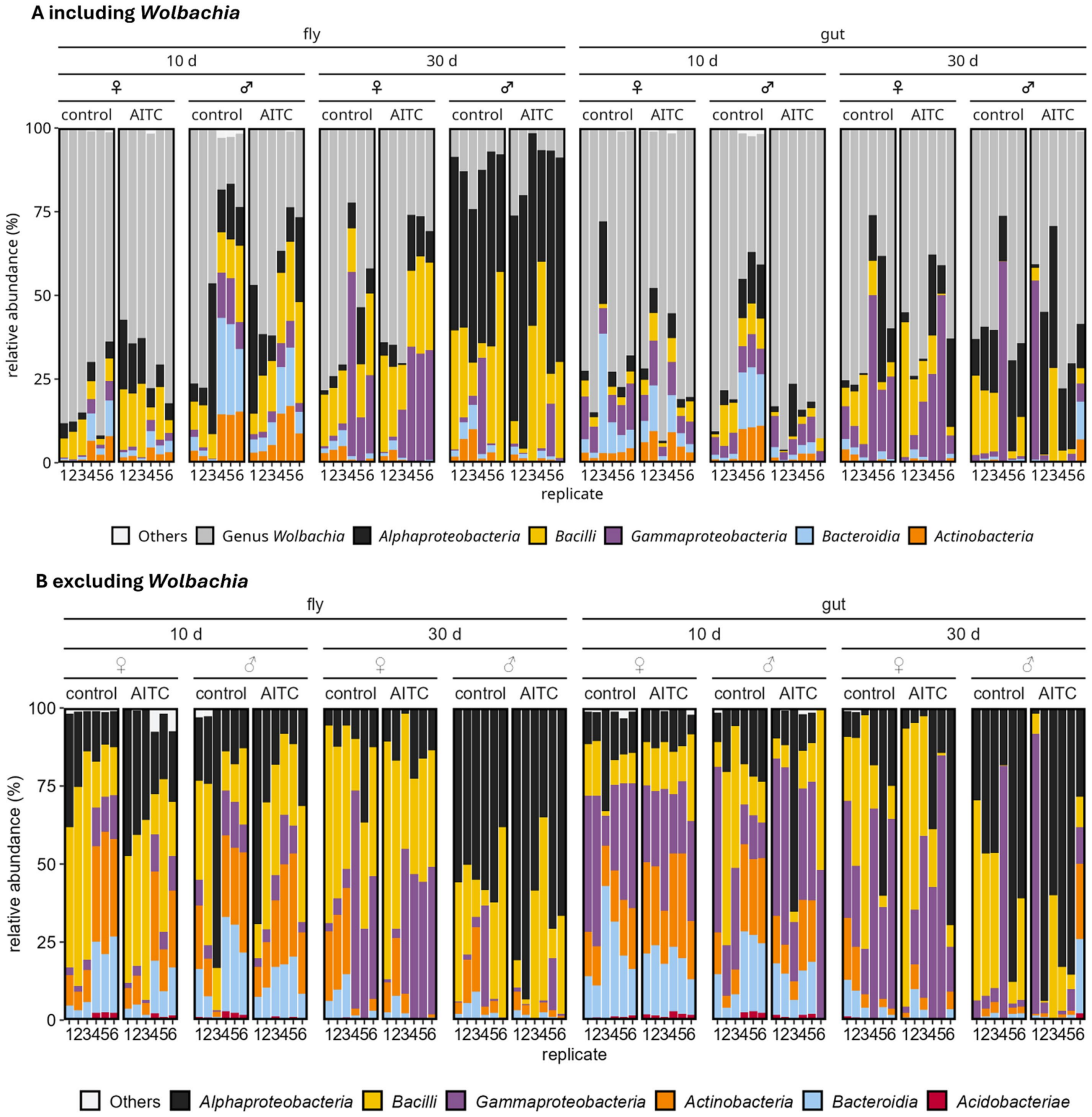
Taxonomic composition of microbial communities at the class level in *Drosophila melanogaster*. Stacked column plot detailing relative abundances of the six most dominant bacterial classes across all 96 samples (unrarefied data) for each replicate. **(A)** Microbial composition including *Wolbachia*; **(B)** microbial composition after excluding *Wolbachia*. Colors represent individual classes, while all remaining 55 classes are grouped as “Others.” Data are shown in corresponding groups, comprising combinations of sample type (whole fly or gut), treatment duration (10 or 30 days), sex [female (♀) or male (♂)], and treatment (control or AITC).

To characterize the body and gut microbiota of *D. melanogaster,* we performed 16S rRNA gene amplicon sequencing on DNA samples of whole individuals and dissected guts to identify bacteria. For all 96 samples, consisting of six replicates per group, we gained a total number of 6,233,116 paired-end reads. After primer clipping, denoising, quality control, merging, and removal of possible chimera 4,133,872 amplicon sequences remained for downstream analysis. The sequences were assigned to 2,709 distinct amplicon sequence variants (ASVs) of which 2,413 remained after removing non-targeted and *Wolbachia* assigned ASVs after classification. ASVs assigned to *Wolbachia* were detected in all 96 samples, as expected because the *D. melanogaster* colony used in this study is *Wolbachia*-positive. *Wolbachia* accounted for a substantial proportion of the total sequence reads (Figure 5A). As *Wolbachia* represents an intracellular endosymbiont with distinct biological characteristic compared to gut microbiota, it was excluded from downstream analyses to allow a more focused interpretation of the remaining bacterial community (Simhadri et al., 2017; Kaur et al., 2021; Porter and Sullivan, 2023). Filtered and unfiltered ASVs are provided in Supplementary Tables S1, S2, respectively. ASV sequence length varied between 269 and 479 bp with a mean of 413 bp. First, we characterized the microbial communities to class and family level. While the main analysis focused on class level taxonomy, additional visualizations at the family level are provided in the Supplementary Figure S3. These data are consistent with the class level patterns and do not indicate clear treatment dependent differences. The classification at class level showed that the microbiota of *D. melanogaster* was dominated by *Alphaproteobacteria* (30.5% relative abundance), *Bacilli* (26.5%), and *Gammaproteobacteria* (20.7%), which together accounted for 77.7% of all ASVs (Figure 5). In these classes the dominant families are *Acetobacteraceae* (27.0% total relative abundance), *Lactobacillaceae* (22.7%), and *Morganellaceae* (9.5%), respectively.

Minor differences were observed between treatment durations and sexes, whereas AITC treatment did not alter the overall class-level composition. Thus, the dominant bacterial community structure remained largely stable across experimental conditions. Variations were noted between 10- and 30-day old flies for both whole *D. melanogaster* and its gut. At the class level, *Acidobacteriae*, *Bacteroidia*, and *Actinobacteria* had a higher relative abundance in 10-day old flies compared to the 30-day old flies. For example, *Bacteroidia* showed a decrease from 7.4 to 1.6%, a 4.6 times lower relative abundance after 30 days of treatment while the relative abundance of class *Bacilli* slightly increased in the 30-days group (from 10.5 to 16.0%, 1.5 times higher). Furthermore, an increase in *Alphaproteobacteria* was observed in male flies and guts in the treatment group of 30 days (from 20.5 to 56.9%, 2.8 times higher), whereas in female flies *Gammaproteobacteria* increased in the treatment group of 30 days (from 6.6 to 28.4%, 4.3 times higher) (Figure 5B, Supplementary Table S1).

Differences of the microbiota at the class level were also present in the 10-days group between whole fly and gut samples in both sexes. Relative abundances in the whole fly were higher than in the fly gut for *Bacilli* (7.6% decreased to 2.9%, 2.7 times lower) and *Alphaproteobacteria* (8.2% decreased to 3.6%, 2.3 times). *Gammaproteobacteria* showed a lower relative abundance in whole flies than in fly guts only (1.9% increased to 5.0%, 2.7 times higher).

In the 30-days group we noted differences between female and male flies in whole fly and gut samples. In female flies we detected a higher relative abundance of *Gammaproteobacteria* (9.5% in females compared to 4.3% in males, 2.2 times lower). We also observed a lower relative abundance of *Alphaproteobacteria* (3.9% in females compared to 14.9% in males, 3.8 times lower) than in the male group (Figure 5, Supplementary Table S1). We could not identify any patterns following a treatment with AITC, neither in whole *D. melanogaster* nor in its guts. However, there seem to be some effects on the microbial composition depending on age and sex.

To better assess differences within our 16S rRNA gene amplicon sequencing data we calculated alpha and beta diversity metrics. For that, we rarefied to 6,802 sequences of which 79 out of 96 samples fulfilled the needed sequencing depth. Per group we retained a minimum of three replicates (Table 1). For all used alpha diversity metrics (Pielou’s evenness, Shannon index, species richness, Faith’s PD) we observed significantly higher values in whole individuals and gut samples for the 10- compared to the 30-days treatment (*p*_KWDadj_ < 0.0013, Kruskal–Wallis with Dunn’s test and BH adjustment). Sex significantly affected Pielou’s evenness (*p*_KWDadj_ = 0.01224) and Shannon index (*p*_KWDadj_ = 0.02340) while other metrics were not changed according to the fly’s sex. We found significant differences in alpha diversity metrics related to treatment duration and sex, but the treatment itself had no impact on the calculated alpha diversity metrics. Beta diversity based on weighted UniFrac distances was assessed using PERMANOVA and revealed no effect of AITC treatment (*p*_PERMANOVA_ = 0.5603, PERMANOVA), while sex significantly influenced microbial community composition in the fly and gut group (both *p*_PERMANOVAadj_ = 0.0007).

**Table 1:** Alpha diversity metrics calculated from rarefied 16S rRNA gene amplicon sequencing data for each experimental group.

Sample type	Treatment duration	Sex	Treatment	*n*	Pielou’s evenness	SD	Species richness	SD	Shannon index	SD	Faith’s PD	SD
Fly	10 days	Female	Control	4	0.65	±0.10	136.25	±55.72	4.59	±1.12	24.81	±6.23
Fly	10 days	Female	AITC	6	0.57	±0.14	141.17	±77.23	4.04	±1.46	34.12	±17.64
Fly	10 days	Male	Control	6	0.59	±0.22	146.83	±50.54	4.27	±1.67	19.90	±12.79
Fly	10 days	Male	AITC	4	0.56	±0.13	109.50	±37.68	3.76	±0.86	14.45	±5.56
Fly	30 days	Female	Control	5	0.52	±0.11	69.60	±39.82	2.93	±0.74	9.43	±5.23
Fly	30 days	Female	AITC	6	0.54	±0.15	37.83	±21.14	2.61	±0.73	5.64	±2.15
Fly	30 days	Male	Control	6	0.38	±0.07	60.00	±29.85	2.20	±0.55	7.10	±2.26
Fly	30 days	Male	AITC	6	0.30	±0.11	38.50	±23.57	1.48	±0.61	6.53	±3.31
Gut	10 days	Female	Control	5	0.71	±0.04	205.40	±46.73	5.46	±0.22	20.75	±7.41
Gut	10 days	Female	AITC	4	0.75	±0.02	163.50	±35.68	5.52	±0.24	15.61	±5.78
Gut	10 days	Male	Control	4	0.59	±0.15	139.75	±54.93	4.17	±1.30	13.62	±3.82
Gut	10 days	Male	AITC	3	0.62	±0.20	182.00	±71.04	4.71	±1.84	19.04	±2.62
Gut	30 days	Female	Control	5	0.45	±0.13	83.20	±71.00	2.77	±1.35	11.78	±8.78
Gut	30 days	Female	AITC	5	0.34	±0.12	62.80	±52.08	1.89	±0.77	9.93	±8.89
Gut	30 days	Male	Control	5	0.31	±0.07	51.00	±32.52	1.65	±0.45	6.39	±1.95
Gut	30 days	Male	AITC	5	0.32	±0.22	55.40	±56.35	1.87	±1.83	6.78	±3.83

Denoted are mean values and standard deviation (SD). Groups are defined by sample type (whole fly or gut), treatment duration (10 or 30 days), sex (female or male), and treatment (control or AITC).

## 4 Discussion

AITC is known to have several effects on human health, including effects on metabolic disorders as well as anti-inflammatory and antioxidant effects (Ahn et al., 2014; Lo et al., 2018; Sahin et al., 2019; Okulicz et al., 2021). It is also known that AITC can alter bacterial growth *in vitro* (Lin et al., 2000; Dufour et al., 2012; Borges et al., 2015; Romeo et al., 2018; Li et al., 2023). Although the microbiota is important for human health also in terms of metabolizing AITC, to the best of our knowledge, no studies have been available so far that investigate the interaction between AITC and the microbiota *in vivo*. Thus, we hypothesized that AITC impacts the microbiota and therefore alters physiology. To address this question, we used *D. melanogaster* as model organism. In contrast to previous studies focusing on infection outcomes in *D. melanogaster* (Zimmermann et al., 2024; Dähn et al., 2026), the present study addresses the effect of AITC under basal conditions. The survival analysis revealed that neither AITC nor the antibiotic treatment, being applied to eradicate the intestinal microbiota, alters the survival rate after 30 days of treatment. The fact, that in our experiments antibiotic treatment did not affect lifespan is in contrast to a previous study, where it could be demonstrated, that in fly embryos an eradication of the microbiota reduced the fly’s lifespan, whereas a bacterial recolonization of the axenic embryos during the first week of adult life increased the lifespan (Brummel et al., 2004). However, several studies showed that axenic flies did not show any differences in lifespan (Ren et al., 2007). Thus, the discussion about the impact of the microbiota on survival and longevity remains controversial. To assess whether AITC treatment affects physiological traits beyond lifespan, we next examined metabolic parameters. No effect of AITC treatment on body weight and glucose content was observed, while a decreased triglyceride content was detected in male flies after AITC treatment for 30 days. This effect persisted even under antibiotic treatment, suggesting that it is not dependent on the microbiota under the conditions tested. The lack of antibiotic effects on triglyceride levels observed in our study contrasts with previous reports (Shin et al., 2011; Newell et al., 2014). However, microbiota-dependent metabolic phenotypes in *D. melanogaster* are known to be highly context-dependent and can vary with host genotype, dietary conditions, and the composition of the microbial community. Differences in these factors may explain the discrepancies between our findings and the existing literature (Shin et al., 2011; Newell et al., 2014; Takada et al., 2025). Although the antibiotic treatment applied in this study has previously been described to substantially reduce the gut microbiota (Staats et al., 2018b), we did not directly verify its effectiveness in our experimental setup. Thus, it should be taken into account, that the persistent reduction of triglyceride levels in male flies under antibiotic treatment may be caused by failure of microbiota elimination. Therefore, future studies should include validation of microbiota depletion, for example by colony-forming unit (CFU) assays or qPCR to ensure microbiota depletion. Based on the previously described effects of AITC on energy metabolism (Ahn et al., 2014; Lo et al., 2018; Sahin et al., 2019; Okulicz et al., 2021), additional changes in body composition would have been expected. One possible explanation for the absence of such effects may be the use of ethanol in the feeding regime, needed to dissolve and administer antibiotics to the feed. In future studies, it would therefore be important to investigate the effects of AITC without the addition of ethanol in axenic flies using alternative methods to AB treatment such as egg bleaching with hypochlorite (Douglas, 2018b). It should also be considered that the number of independent experiments in this study was relatively small (*n* = 3), which may have limited statistical power. Consequently, subtle effects may not have been detected. While AITC treatment exhibited no effect in females, it led to a significant reduction of triglyceride levels in male flies pointing towards sex-specific effects of AITC. We have already demonstrated sex-specific differences in gene expression in key regulators of energy metabolism (Dähn and Wagner, 2025). This concerns among others *Akh* (*Adipokinetic hormone*) and *AkhR* (*Adipokinetic hormone receptor*), which are higher expressed in male flies. It is therefore conceivable that these inherent sex-specific differences in energy metabolism may underly the observed divergent effects of AITC between female and male flies. To verify this hypothesis, experiments using transgenic flies with a targeted knock-out or overexpression of *Akh* or *AkhR* would be required.

Although AITC affected triglyceride levels in males, no changes in lifespan were detected. This is surprising, as it is documented that in *D. melanogaster* survival is negatively correlated with triglyceride levels (Skorupa et al., 2008; Katewa et al., 2016; Chauhan et al., 2021; Yuan et al., 2024). A reason may be that in the present study survival was only monitored over a period of 30 days as an effect might only become apparent after a longer observation period (until all flies have died).

Moreover, it is important to consider the composition of the gut microbiota in this context. In this study, the dominant bacterial classes in the fly gut microbiota have been *Alphaproteobacteria*, *Bacilli*, *Gammaproteobacteria*, and *Actinobacteria,* regardless of treatment. Although it is well documented that the gut microbiota of *D. melanogaster* varies between different strains and is highly modulated by diet, these classes have been demonstrated to be the ones mostly present in the gut microbiota of *D. melanogaster* (Broderick and Lemaitre, 2012; Staubach et al., 2013; Early et al., 2017; Douglas, 2018a; Garcia-Lozano et al., 2020). While it is known that AITC alters bacterial growth *in vitro* (Lin et al., 2000; Dufour et al., 2012; Romeo et al., 2018; Li et al., 2023), dietary AITC treatment had no significant effects on the composition or diversity of the *D. melanogaster* microbiota in our study. Neither alpha nor beta diversity analyses revealed any treatment effects in whole flies or gut samples, which may refer to the fact that the chosen AITC concentration might have been too low for antimicrobial effects. For example, a minimum inhibitory concentration (MIC) of 100 μg/mL (this is approximately 1 mM) AITC was needed to inhibit bacterial growth of *E. coli*, *L. monocytogenes*, *P. aeruginosa* and *S. aureus in vitro* (Borges et al., 2015). In this study, the flies were fed a diet enriched with AITC containing only a quarter (0.25 mM) of this concentration, as higher concentrations were not accepted by the flies. Therefore, the absence of detectable microbiota-related effects may be due to insufficient exposure levels of AITC. As our findings are limited to the maximum tolerated AITC concentrations in the diet of the flies, our results may not reflect the effects at higher, potentially antimicrobial concentrations. Additionally, it should be taken into account that isothiocyanates can be metabolized, for example via conjugation with glutathione (Narra et al., 2025). Metabolites resulting from AITC may be less effective against intestinal bacteria which could explain the lack of results. Although it has been shown that AITC exhibits antimicrobial activity against certain bacteria (Lin et al., 2000; Dufour et al., 2012; Romeo et al., 2018; Li et al., 2023), it may be possible that the intestinal bacteria in *D. melanogaster* are tolerant against this compound. To test this, the fly’s gut microbiota needs to be cultured *ex vivo* to examine the anti-microbial effects of AITC *in vitro*. However, although no compositional changes in the microbiota were detected, AITC may still modulate bacterial metabolism, as it is able to react with, for example, thioredoxin reductase and acetate kinase, and in consequence may affects membrane integrity (Brown and Hampton, 2011; Borges et al., 2015). These functional shifts would not necessarily manifest as changes in relative taxonomic abundance but may affect metabolite profiles or other microbial contributions to affect host physiology. This hypothesis should be tested *in vitro*, ideally through an *ex vivo* cultivation of the fly’s gut microbiota. Instead, significant differences between sex, age and sample type (gut or whole body) were observed. While there were no detectable effects of AITC treatment on the fly microbiota, we demonstrated an age-dependent decrease in alpha diversity between 10- and 30-day old flies. This observation is consistent with previous findings showing significant age-related changes in the gut microbiota of *D. melanogaster* (Han et al., 2017). One reason for age-dependent changes may be a dysregulation of Rel/NFkB signaling (Guo et al., 2014): in an aging intestine a chronic activation of the transcription factor Foxo occurs resulting in a dysregulation of Rel/NFkB, which in turn may cause commensal dysbiosis, stem cell hyperproliferation and epithelial dysplasia. Dysbiosis has been associated with an increase in *Alphaproteobacteria* and *Gammaproteobacteria* (Clark et al., 2015), which is in line with our data that also reveal an increase in *Alphaproteobacteria* in the older male flies and guts, along with a rise in *Gammaproteobacteria* in 30-day old female flies. These findings highlight the complex changes in the microbiota related to age and sex, which may have further implications for understanding microbial dynamics and their impact on health. However, a limitation of the present microbial community analysis is given by the fact that the applied amplicon sequencing approach did not permit confident taxonomic interpretation lower than family level. Thus, the analysis focused on higher taxonomic ranks. In addition, it should be noted that our data excluded *Wolbachia* assigned ASVs from further analysis. While *Wolbachia* has been reported to influence the hosts physiology and may also interact with other members of the gut microbiota, it represents a maternally inherited intracellular endosymbiont that resides within host cells and differs fundamentally from the gut microbiota (Simhadri et al., 2017; Kaur et al., 2021; Porter and Sullivan, 2023). In contrast to the transient and environmentally acquired gut microbiota of *D. melanogaster*, which is largely shaped by dietary intake, *Wolbachia* is vertically transmitted and occupies intracellular niches. Therefore, it was excluded from downstream analyses to allow a more focused assessment of the gut-associated bacterial community. Due to its high relative abundance and distinct biology, inclusion of *Wolbachia* may mask variation in the environmentally acquired microbiota and complicate ecological interpretation. It should also be considered that AITC is a reactive and volatile compound, and its stability in the diet over time may influence effective exposure. Although flies were transferred to fresh feed every 2–3 days, we did not directly assess AITC stability or feeding behavior in the present study. In previous work, we observed reduced feed intake in male flies upon AITC supplementation under comparable conditions (Zimmermann et al., 2024). However, as these experiments were performed in a different experimental context and without antibiotic treatment, it remains unclear to what extent these results are transferable to the present study. Therefore, differences in compound availability or intake cannot be excluded. Overall, the present study did not show consistent effects of AITC treatment in *D. melanogaster* neither on physiological parameters tested nor on the microbiota composition. However, a reduction in triglyceride levels was observed in male flies after prolonged AITC exposure which persisted under antibiotic treatment. Importantly, the absence of changes in relative microbiota composition does not exclude potential effects on total bacterial load, viability, or metabolic activity. Therefore, microbiota-related effects cannot be fully excluded. Nonetheless, we identified sex and age as potential drivers of the microbial composition. Our study has also identified several areas for future experiments including (a) verifying the efficiency of antibiotic treatment in eliminating the fly’s microbiota; (b) exploring alternative approaches for generating axenic flies, such as embryo bleaching, to establish a more robust and reliable axenic model; (c) culturing dominant bacterial taxa and assessing the effects of AITC on bacterial survival and metabolism *in vitro* to determine direct microbial sensitivity and to identify an appropriate, physiologically relevant dosage; and (d) subsequently examining the effects of AITC on the absolute abundance of viable microbiota or specific bacterial groups *in vivo* using microbial cell counts.

## Data Availability

The datasets presented in this study can be found in online repositories. The names of the repository/repositories and accession number(s) can be found at: https://www.ebi.ac.uk/ena/browser/view/PRJEB100584.
