# Supplementary material for: Dietary allyl-isothiocyanate affects male triglyceride levels in Drosophila melanogaster without detectable changes in microbiota composition
Source: Front Microbiol. 2026 May 13;17:1817451. doi: 10.3389/fmicb.2026.1817451 (PMC13212214; doi:10.3389/fmicb.2026.1817451)
Supplement: Supplementary file 2 [file Data_Sheet_2.pdf]

Supplementary Material

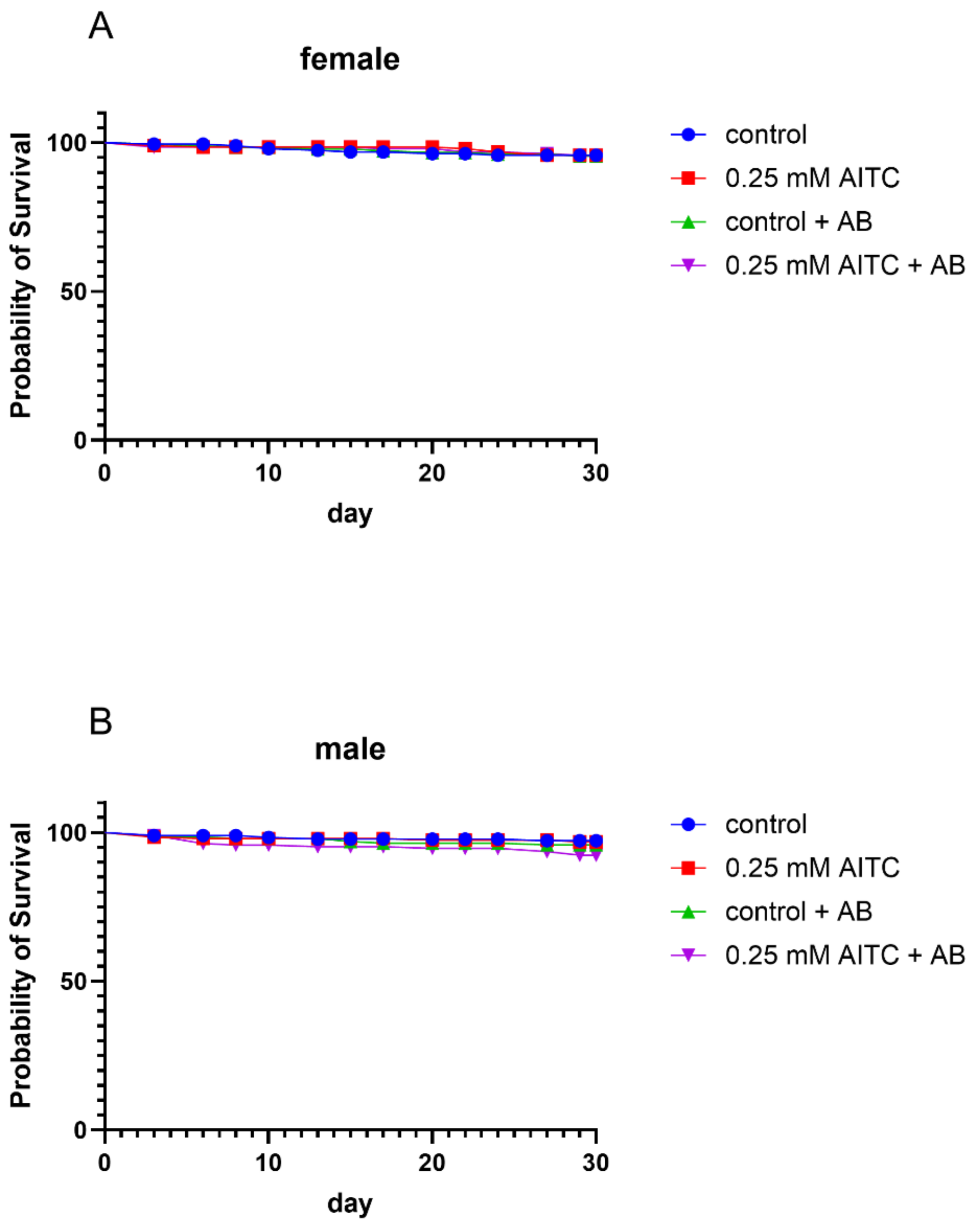

Figure S1: Survival curves of female (A) and male (B) *Drosophila melanogaster*, treated with or without 0.25 mM AITC and antibiotics (AB,). Flies were maintained under standard conditions and subjected to either control diet, 0.25 mM AITC, antibiotic treatment (AB: 500 µg/ml ampicillin, 50 µg/ml tetracycline, and 200 µg/ml rifamycin), or a combination of both (0.25 mM AITC + AB).

Results show three independent experiments; each consisted of 75 flies per treatment. Significant differences between treatments were tested by the Kaplan–Meier approach and a log-rank test. Significance was accepted at $p < 0.05$.

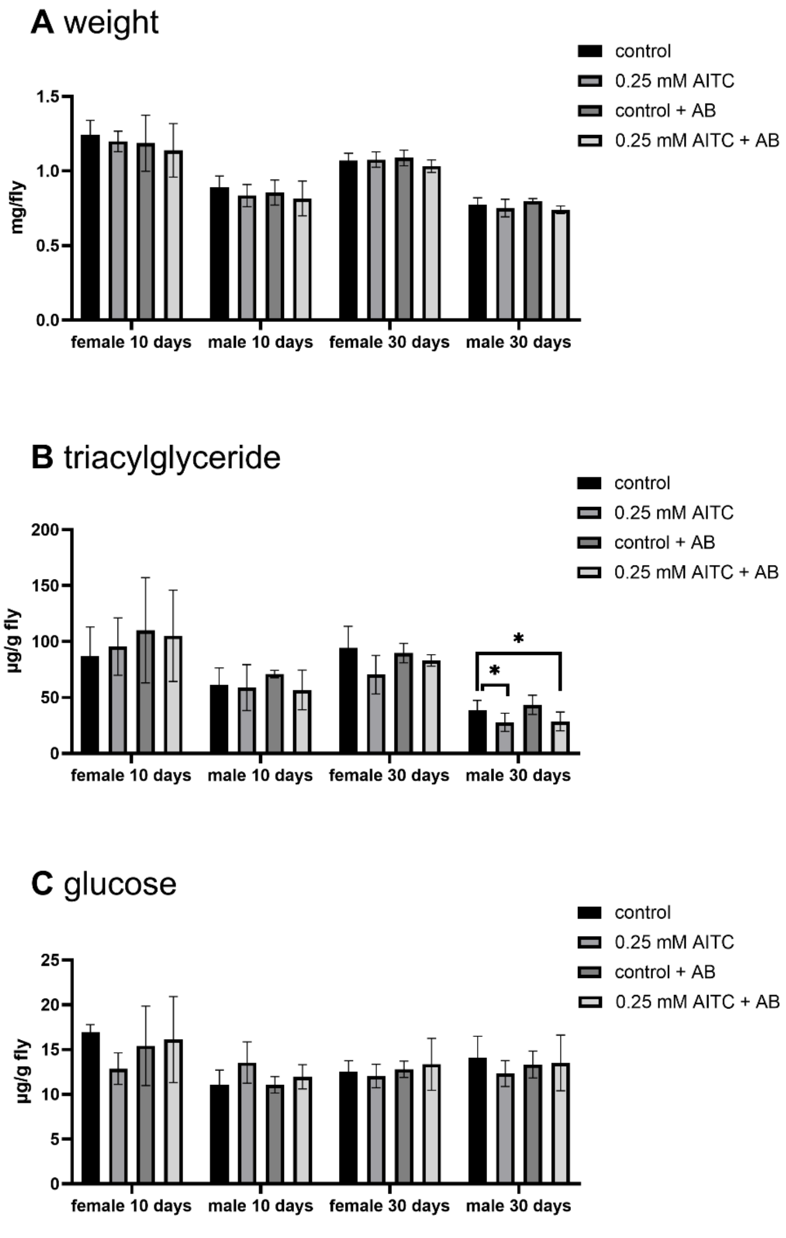

Figure S2: Weight (A), triacyl glyceride content (B) and glucose content (C) of female and male *Drosophila melanogaster*, after 10 and 30 days of treatment with or without 0.25 mM AITC and antibiotics (AB). Flies were maintained under standard conditions and subjected to either control diet, 0.25 mM AITC, antibiotic treatment (AB: 500 µg/ml ampicillin, 50 µg/ml tetracycline, and 200 µg/ml rifamycin), or a combination of both (0.25 mM AITC + AB). Body weight was normalized to the respective control group. Data are presented as mean + SD of the three independent

experiments (n=3), each consisting of 5 flies. Statistical analysis was performed using a one-way ANOVA followed by Dunnett's multiple comparison post hoc test.

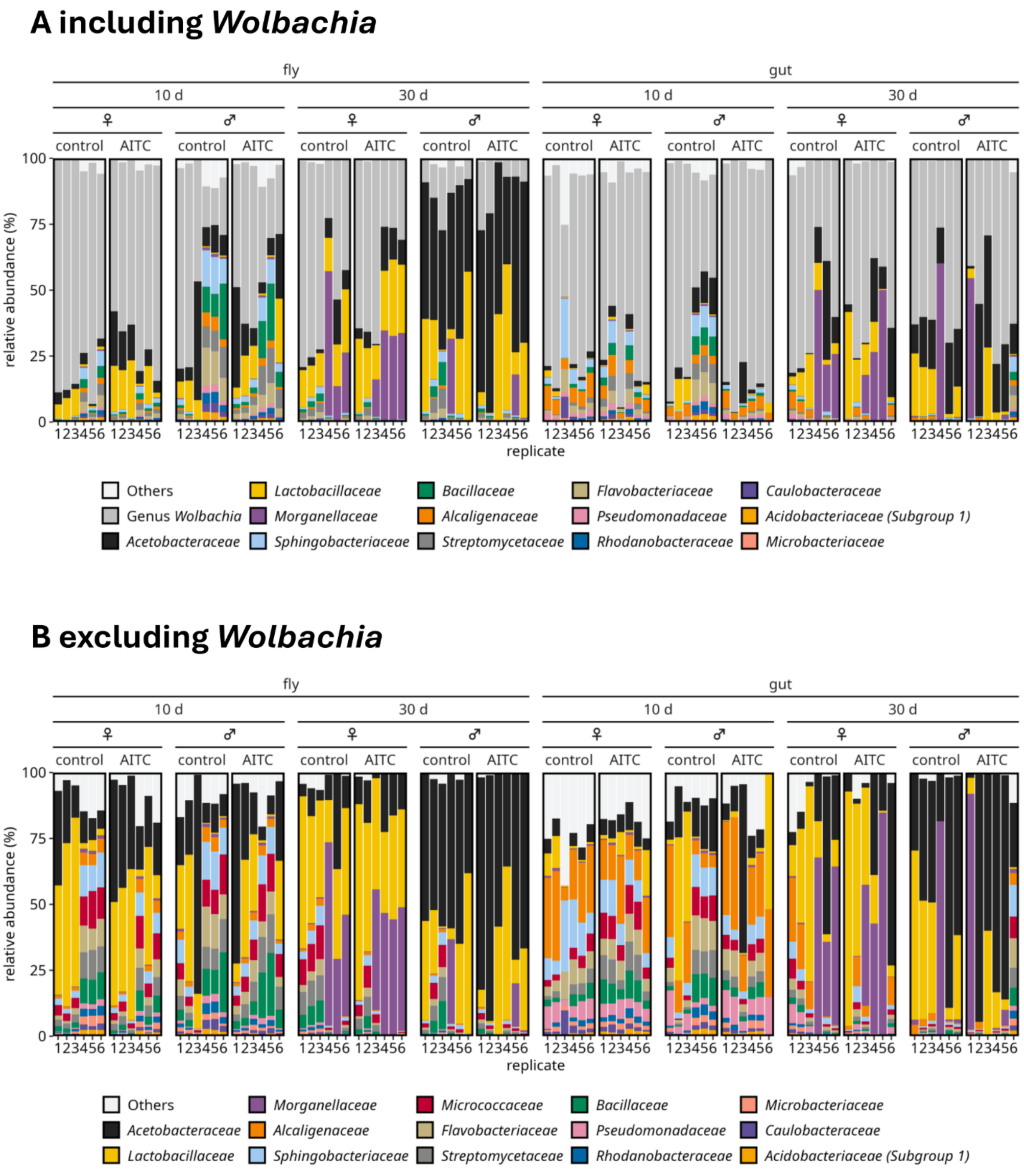

Figure S3: Taxonomic composition of microbial communities at the family level in *Drosophila melanogaster*. Stacked column plot detailing relative abundances of the six most dominant bacterial classes across all 96 samples (unrarefied data) for each replicate. A: Microbial composition including

Wolbachia; B: Microbial composition after excluding *Wolbachia*. Colors represent individual classes, while all remaining 55 classes are grouped as "Others". Data are shown in corresponding groups, comprising combinations of sample type (whole fly or gut), treatment duration (10 or 30 days), sex (female (♀) or male (♂)), and treatment (control or AITC).
